# Heart rate variability and target organ damage in hypertensive patients

**DOI:** 10.1186/1471-2261-12-105

**Published:** 2012-11-15

**Authors:** Paolo Melillo, Raffaele Izzo, Nicola De Luca, Leandro Pecchia

**Affiliations:** 1 Multidisciplinary Department of Medical Sciences, Second University of Naples, Via Pansini, 5, Naples, Italy; 2 Departments of Clinical Medicine, Cardiovascular and Immunological Sciences, Federico II University Hospital, Via Pansini, 5, Naples, Italy; 3 Electrical Systems and optics research division, Faculty of Engineering, University of Nottingham, University Park, Nottingham, NG7 2RD, UK

**Keywords:** Heart Rate Variability, Target organ damage, Hypertension

## Abstract

**Background:**

We evaluated the association between linear standard Heart Rate Variability (HRV) measures and vascular, renal and cardiac target organ damage (TOD).

**Methods:**

A retrospective analysis was performed including 200 patients registered in the Regione Campania network (aged 62.4 ± 12, male 64%). HRV analysis was performed by 24-h holter ECG. Renal damage was assessed by estimated glomerular filtration rate (eGFR), vascular damage by carotid intima-media thickness (IMT), and cardiac damage by left ventricular mass index.

**Results:**

Significantly lower values of the ratio of low to high frequency power (LF/HF) were found in the patients with moderate or severe eGFR (p-value < 0.001). Similarly, depressed values of indexes of the overall autonomic modulation on heart were found in patients with plaque compared to those with a normal IMT (p-value <0.05). These associations remained significant after adjustment for other factors known to contribute to the development of target organ damage, such as age. Moreover, depressed LF/HF was found also in patients with left ventricular hypertrophy but this association was not significant after adjustment for other factors.

**Conclusions:**

Depressed HRV appeared to be associated with vascular and renal TOD, suggesting the involvement of autonomic imbalance in the TOD. However, as the mechanisms by which abnormal autonomic balance may lead to TOD, and, particularly, to renal organ damage are not clearly known, further prospective studies with longitudinal design are needed to determine the association between HRV and the development of TOD.

## Background

Cardiovascular (CV) diseases are one of the most leading causes of morbidity and mortality in high developed countries [1]. A number of prospective population or cohort studies, such as the Atherosclerosis Risk in Communities Study [2], the Rotterdam Study [3], the Cardiovascular Health Study [4], and the Carotid Atherosclerosis Progression Study [5], have shown that asymptomatic organ damage is significantly related to incident CV events. If CV involvement is early detected by physicians, it is possible to influence the progression or regression of the disease by the therapy [6]. Although there is still a little information on the specific causes of these pathologies, a recent review by Thayer suggested that autonomic imbalance may be a final common pathway to increased morbidity and mortality from a host of conditions and pathologies, including CV diseases [7]. Analysis of heart rate variability (HRV) on the basis of routine 24-hour Holter recordings has been shown to provide a sensitive measurement of cardiac control by the autonomous nervous system (ANS) [8,9]. HRV is a non-invasive measure reflecting the variation over time of the period between consecutive heartbeats (RR intervals) [8]. In fact, heart rate (HR), which continuously fluctuates over time, is under the influence of control mechanisms aimed at maintaining a dynamic stability called homoeostasis [10]. In this equilibrium, the sympathetic stimulation causes acceleration in HR by increasing the firing rate of pacemaker cells in the heart’s sino-atrial node, while the parasympathetic system causes deceleration in HR by decreasing of the firing rate of pacemaker cells [8]. Clinical studies have shown reduced HRV in patients with congestive heart failure [11-16], diabetes [17], and white coat hypertension [18]. Moreover, decreased HRV measures have been shown to be a risk factor for mortality in chronic haemodialysis patients [19] and for progression to end-stage renal disease [20,21]. Furthermore, several studies have shown that incidence of CV events can be reduced by therapeutic correction of any modifiable risk factors, such as hypertension, abnormal cholesterol, diabetes, smoking habit or physical inactivity. As noted and shown in the review by Thayer [7], there is at least some data to suggest that each of these risk factors is associated with decreased HRV. Regarding hypertensive patients a sympatho-vagal imbalance as evaluated by HRV with increased sympathetic activity and reduced vagal tone has been reported [22] and a recent paper by Garcia-Garcia [23] investigate the correlation between heart rate and the parameters that assess vascular, renal and cardiac target organ damage (TOD). However, only two HRV measures were selected in the study by Garcia-Garcia [23]. The aim of the current study was to evaluate the association between linear standard HRV measures with the vascular, renal and cardiac TOD in hypertensive patients registered in the Campania Salute network. The Campania Salute network is an open registry collecting information from a network of general practitioners and community hospitals networked with the centre, and providing a centralised database including demographics and clinical information of the patients.

## Methods

### Population study

For the present study, among the initial cohort of 12,000 patients registered in the database of the Campania Salute Network, we selected all the hypertensive subjects who underwent at least one visit in the Outpatient Hypertension Clinic of the University of Naples “Federico II” from 2000 to 2010 and were evaluated by a cardiac and carotid ultrasonography and by a 24h Holter ECG. The ECG Holter was performed after a one-month antihypertensive therapy wash-out. Details on this cohort have been previously reported [24,25]. Exclusion criteria for the present analysis were: diagnosis of secondary resistant and/or uncontrolled hypertension, prevalent CV disease, clinical history of cancer, liver cirrhosis and/or failure, narcotics abuse, lifestyle changes in the last 12 months. Moreover, patients with atrial fibrillation and frequent ectopic beats as assessed by Holter were excluded. Prevalent CV disease was defined as history of previous myocardial infarction or angina or procedures of coronary revascularization, stroke or transitory ischemic attack, congestive heart failure, chronic kidney disease more than grade 3 (eGFR by MDRD < 30mL/min/1.73m^2^) at the time of the first examination in the outpatient clinic. Prevalent CV disease was excluded by an ad-hoc committee in the Hypertension Centre, based on patients’ history, contact with the referring general practitioner and clinical records documenting the occurrence of disease. In the sub-cohort of 10254 patients with arterial hypertension, 4257 patients were excluded for diagnosis of secondary resistant and/or uncontrolled hypertension, prevalent CV disease, clinical history of cancer, liver cirrhosis and/or failure, narcotics abuse, lifestyle changes in the last 12 months; 1814 for chronic kidney disease more than grade 3 (eGFR by MDRD < 30mL/min/1.73m^2^); 2556 for missing data (i.e. ultrasonography, holter ECG); 942 for atrial fibrillation and 485 for frequent ectopic beats.

### Ethical issues

All the data of the patients (i.e. medical history, physical examination, routine laboratory tests and other diagnostic procedures) were stored in the computerised database of Campania Salute Network. The database generation of the Campania Salute Network was approved by the Federico II University Hospital Ethic Committee. All the participants signed informed consent to use data for scientific purposes.

### Protocol

At the first visit all patients were given a detailed questionnaire inquiring about specifics lifestyle behaviours and smoking habit. In the current study they were categorised as non-smokers, ex-smokers or smokers. During the visits, blood pressure (BP), lipid and glucose profiles were measured for each patient by standard methods. Diagnosis and stratification of essential hypertension was performed according to the criteria established by the Guidelines for the Management of Arterial Hypertension [26]: systolic and diastolic BP were measured by a standard aneroid sphygmomanometer after 5 min rest in the supine position, according to the current guidelines [26]. Three BP measurements were obtained at 2-min intervals. The averages of these measurements were used for the analysis. Glomerular filtration rate (eGFR) was estimated by the Modification of Diet in Renal Disease (MDRD) formula [27]. Diabetes was defined as a fasting blood glucose ≥126 mg/dL or active glucose-lowering therapy [28].

### Echocardiography

Two-dimensional-guided M-mode echocardiograms were performed using a dedicated ultrasound machine (SONOS 5500, Philips) with an ultrasound transducer of 2.5 MHz. The examinations were recorded on a digital recorder and analysed by three independent, trained and experienced physicians. The parameters relative to the left ventricle (LV) were measured in the parasternal long-axis view and obtained, according to the criteria of the American Society of Echocardiography [29], as an average of at least three measurements, as also performed in previous studies [30,31]. LV mass was determined by using the formula developed by Devereux [32] as recommend by American Society of Echocardiography (ASE) [33] and divided by the body surface area to calculate LV mass index (LVMi, g/m^2^). Intraoperator and interassay variability were 5% and 6%, respectively [34].

### Carotid ultrasound

B-mode ultrasonography of carotid arteries was performed with patients in the supine position with the neck extended in mild rotation. The scanning protocol was performed with an ultrasound device (SONOS 5500, Philips) equipped with a 7.5-MHz high-resolution transducer with an axial resolution of 0.1 nm. Examinations were recorded on S-VHS videotapes. All measurement were analysed by three different trained experienced physicians. An average of two readings was considered for subsequent calculations. The accuracy of determinations was evaluated as previously described by Lembo et al. [35]: the variability of measurements to evaluate intrasonographer and intersonographer reproducibility was 0.01 and 0.03 mm, respectively. The maximum arterial intima media thickness (IMT) in up to 12 arterial walls, including the right and the left, near and far distal common carotid (1 cm), bifurcation, and proximal internal carotid artery was estimated offline with an image processing workstation with the software COMPACS (Rev. 10.5.8, Medimatic, Genoa, Italy).

### Assessment of TOD

Cardiac Involvement was evaluated as Left Ventricular Hypertrophy (LVH) which was diagnosed if LVMi exceeded 110 g/m^2^ in female and 125 g/m^2^ in male [36]. Vascular involvement was assessed as carotid artery atherosclerosis shown by increased IMT in B-mode ultrasonography. IMT values between 1.0 and 1.3 mm were defined as "thickening" and those higher than 1.3 mm as “plaque”. Chronic kidney disease was assessed by eGFR and involvement was quantified as follows:

1. group 1: increased or normal eGFR (eGFR ≥ 90 mL/min/1.73 m^2^)

2. group 2: mild eGFR (60 < eGFR < 90 mL/min/1.73 m^2^)

3. group 3: moderate eGFR (eGFR ≤ 60 mL/min/1.73 m^2^) [37]

### Processing 24-hour holter recordings

On 2 consecutive days, patients underwent a 24-hour ECG Holter recording. The recorders were applied between 9 and 11 AM on a working day, and the patients were asked to follow as closely as possible their usual daily activities during each monitoring session. They were asked to stay in bed from 11 PM to 7 AM, and all reported to have slept normally during the nights they were monitored. The series of normal to normal (NN) beat intervals were obtained from ECG recordings using OSAS, an open-source software for QRS detection and beat classification [38]. Standard long-term HRV analysis on nominal 24-h recordings according to International Guidelines was performed [8]. The HRV analysis was performed using PhysioNet's HRV Toolkit [39]. We chose this toolkit as it is an open source and a rigorously validated package. All the computed basic time- and frequency-domain HRV measures were widely used in the literature [8]. A number of standard statistical time-domain HRV measures are calculated: Average of all NN intervals (AVNN), standard deviation of all NN intervals (SDNN), standard deviation of the averages of NN intervals in all 5-min segments of a 24-h recording (SDANN), mean of the standard deviations of NN intervals in all 5-min segments of a 24-h recording (SDNN IDX), square root of the mean of the sum of the squares of differences between adjacent NN intervals (RMSSD), percentage of differences between adjacent NN intervals that are longer than 50 ms (pNN50). The frequency-domain HRV measures rely on the estimation of power spectral density (PSD) computed, in this work, by Lomb-Scamble periodogram [40]. After PSD estimation, six standard frequency-domain HRV measures were calculated: total spectral power of all NN intervals up to 0.4 Hz (TOTPWR), between 0 and 0.003 Hz (ULF), between 0.003 and 0.04 Hz (VLF), between 0.04 and 0.15 Hz (LF), and between 0.15 and 0.4 Hz (HF), ratio of low to high frequency power (LF/HF).

### Statistical analysis

Data were analysed by the use of PASW Statistics 18 software (Release 18.0; SPSS IBM, Chicago, IL, USA). Univariate differences were analysed using Kruskal-Wallis and Wilcoxon test for HRV measures, ANOVA and t-test for the other continuous variables (for instance age, IMT, etc.) and χ^2^ test for the categorical variables (for instance sex, smoking). For each HRV measure, which differs significantly among the three groups, an adjusted model was proposed by performing a binary or multinomial logistic regression, as appropriate. For each factor or covariate, the coefficient of the estimated regression model (β), the corresponding statistical significance (p), the odds ratio (OR) and the confidence interval for OR at 95% are presented. A p-value less than 5% was considered statistically significant.

## Results

200 patients were analysed (127 male and 73 female). Demographic, clinical and laboratory characteristics of the study sample are shown in Table 1. Table 2 shows the characteristic of the study sample of patients categorised by eGFR. The moderate eGFR group is significantly older than the others and has a significantly higher proportion of patients taking diuretics. Systolic BP and Pulse Pressure values were significantly higher in mild decreased eGFR group (compared to normal eGFR group), where IMT values were significantly lower in normal eGFR group (compared to moderate decreased eGFR group). Table 3 shows the descriptive statistics of HRV measures in the groups stratified by eGFR. The three groups differed significantly in LF/HF. Table 4 shows the characteristic of the study sample of patients categorised by IMT. The Plaque group is significantly older than the others. LVMi values were significantly lower in the normal group compared to the Plaque group and the proportion of patients with LVH was significantly lower. A significantly higher proportion of patients with family history of hypertension was assessed in the Thickening group. A significant difference in the proportion of patients taking beta-blockers was observed. Table 5 shows the descriptive statistics of HRV measures in the three groups according to IMT. The three groups differed significantly in SDNN, SDANN, SDNN IDX TOTPWR, LF, and LF/HF. Table 6 shows the characteristic of the study sample of patients categorised by LVH. The LVH group is significantly older than the group without LVH. IMT values were significantly higher in the LVH group and the proportion of patients with vascular abnormalities was significantly higher. A significantly higher proportion of patients taking diuretics was assessed in LVH group. The values of Systolic BP and Pulse Pressure were significantly higher in the LVH group. Table 7 shows the comparison of HRV measures in the patients with and without LVH. The two groups differed significantly in LF/HF. Most differences persisted even in the adjusted models, which are reported in Table 8. As concerns renal TOD, the multinomial logistic regression selected age, family history of hypertension and systolic BP. Higher values of LF/HF are associated with an increased probability that a subject belongs to normal or mild eGFR groups rather than to moderate group (OR 2.718 and 2.637, respectively). Older age is associated with a decreased probability of being in normal or mild decreased eGFR groups (OR 0.897 and 0.943, respectively). The absence of family history of hypertension is associated with an increased probability of belonging to normal eGFR group (OR 2.951). Elevated systolic BP seems to be associated with a slightly increased probability of belonging to mild decreased eGFR group (OR 1.024). As regards vascular TOD, the adjusted models confirmed the differences in the values of SDNN and SDANN. In fact, higher values of SDNN and SDANN are associated with an increased probability that a subject had no vascular alterations rather than plaque. Moreover, this model confirms that older age is associated with vascular alterations. As regards cardiac TOD, the binomial logistic regression did not confirm the difference in LF/HF and showed that other variables are associated with LVH such as age, systolic BP, cholesterol, and diuretics.

**Table 1:** Characteristics of the study sample of patients

**Characteristic**	**Value**
Age (years)	62.5 ± 12.1
Sex (male/female, %)	63.5/36.5
Family history of hypertension (yes/no, %)	56.5/43.5
Family history of stroke (yes/no, %)	19.5/80.5
Smokers (yes/ex/no, %)	17.5/21/61.5
Diabetes (yes/no, %)	10/90
Diastolic BP (mmHg)	75.6 ± 12.0
Systolic BP (mmHg)	133.2 ± 22.6
Pulse pressure (mmHg)	57.6 ± 17.8
Fasting blood glucose (mmHg)	102.7 ± 24.1
Total Cholesterol (mg/dl)	185.8 ± 40.6
BMI (kg/m^2^)	27.7 ± 4.6
Beta-blockers (yes/no, %)	33.5/66.5
Alphabeta-blockers (yes/no, %)	10.5/89.5
Alpha-blockers (yes/no, %)	8/92
Diuretics (yes/no, %)	43.5/56.5
ACE inhibitor (yes/no, %)	37.5/62.5
Dihydropyridine (yes/no, %)	26/74
eGFR (mL/min/1.73 m^2^)	77.0 ± 18.9
Kidney Involvement (group 1/ 2 /3 %)	24/59.5/16.5
IMT (mm)	2.24 ± 1.56
Vascular abnormalities (no/ thickening/plaque, %)	13/11/78
LVMi g/m^2^	130.5 ± 31.0
Left Ventricular hypertrophy (no/yes, %)	40/60

Data are expressed as mean and standard deviation for continuous variables (i.e. age) and as percentage of patients per each group for categorical variables (i.e. gender).

**Table 2:** Characteristics of the study sample of patients stratified according to the eGFR

	**Normal eGFR (group 1)**	**Mild decreased eGFR (group 2)**	**Moderate decreased eGFR (group 3)**	**P Values**			
				**ANOVA or χ**^**2**^	**group 1 vs group 2**	**group 1 vs group 3**	**group 2 vs group 3**
Age (years)	56 ± 11.5	63,0 ± 11.7	70.0 ± 9.4	**<0.001**	**0.001**	**<0.001**	**0.05**
Gender (male/female)	31/17	76/43	20/13	0.928			
Family history of hypertension (yes/no)	25/23	69/50	19/14	0.778			
Family history of stroke (yes/no)	11/37	22/97	6/27	0.790			
Smokers (yes/ex/no)	13/8/27	8/27/7	27/75/21	0.379			
Diabetes (yes/no)	5/43	11/108	4/29	0.883			
Diastolic BP (mmHg)	73.2 ± 14.0	77.3 ± 11.5	73.0 ± 9.8	0.048	0.129	1	0.185
Systolic BP (mmHg)	124.5 ± 23.3	137.5 ± 20.0	130.2 ± 27.3	**0.002**	**0.002**	0.754	0.286
Pulse pressure (mmHg)	51.3 ± 14.1	60.1 ± 16.9	57.3 ± 23.3	**0.014**	**0.010**	0.396	1
Fasting blood glucose (mmHg)	99.7 ± 32.2	102.8 ± 20.0	106.7 ± 23.9	0.432	1	0.589	1
Total Cholesterol (mg/dl)	178.9 ± 36.4	187.7 ± 40.8	189.0 ± 45.8	0.395	0.610	0.817	1
BMI (kg/m^2^)	27.4 ± 5.2	28.1 ± 4.6	26.7 ± 3.5	0.261	1	1	0.379
Beta-blockers (yes/no)	15/33	41/78	11/22	0.924			
Alphabeta-blockers (yes/no)	5/43	14/105	2/31	0.639			
Alpha-blockers (yes/no)	3/45	8/111	5/28	0.252			
Diuretics (yes/no)	17/31	49/70	21/12	**0.03**			
ACE inhibitor (yes/no)	16/32	48/71	11/22	0.604			
Dihydropyridine (yes/no)	12/36	30/89	10/23	0.826			
eGFR (mL/min/1.73 m^2^)	101.9 ± 12.0	74.3 ± 8.8	50.6 ± 8.0	**<0.001**	**<0.001**	**<0.001**	**<0.001**
IMT (mm)	1.8 ± 0.76	2.23 ± 1.21	2.91 ± 2.85	**0.007**	0.282	**0.005**	0.81
Vascular abnormalities (no/ thickening/plaque)	33/6/9	91/13/15	28/3/2	0.502			
LVMi g/m^2^	124.3 ± 26. 2	133.0 ± 32.3	130.4 ± 32.1	0.258	0.301	1	1
Left Ventricular hypertrophy (no/yes)	24/24	44/75	12/21	0.268			

Data are expressed as mean and standard deviation for continuous variables (i.e. age) and as number of patients per each group for categorical variables (i.e. gender).

The p-values refer to ANOVA and post-hoc comparisons with Bonferroni corrections for continuous variables, to χ^2^ for categorical variables.

**Table 3:** Comparisons of HRV measurement in patients stratified by eGFR

	**Normal eGFR (group 1)**					**Mild decreased eGFR (group 2)**					**Moderate decreased eGFR (group 3)**					**P-values**
	**Mean**	**St. Dev.**	**Quartiles**			**Mean**	**St. Dev.**	**Quartiles**			**Mean**	**St. Dev.**	**Quartiles**			
			**1**^**st**^	**2**^**nd**^	**3**^**rd**^			**1**^**st**^	**2**^**nd**^	**3**^**rd**^			**1**^**st**^	**2**^**nd**^	**3**^**rd**^	
AVNN	859.7	109.4	784.1	848.9	916.8	867.8	136.4	774.9	853.5	954.4	884.4	119.0	793.6	875.4	963.4	0.525
SDNN	125.7	30.2	101.9	119.5	146.1	119.4	38.8	92.2	111.1	140.1	116.6	36.1	94.7	113.6	141.1	0.280
SDANN	113.4	32.5	90.1	108.6	137.0	106.6	38.7	78.0	99.4	129.1	105.2	36.1	81.2	104.0	132.4	0.308
SDNN IDX	51.9	11.8	43.2	51.4	58.9	51.2	16.5	40.7	47.4	61.1	45.4	13.7	36.2	44.3	58.3	0.168
RMSSD	32.6	12.5	24.4	30.1	38.1	35.7	18.9	22.6	30.7	42.3	35.6	14.3	24.4	33.5	42.1	0.602
pNN50	8.8	6.6	3.8	7.7	11.9	11.2	10.4	2.8	7.9	17.8	11.5	11.0	4.1	9.7	12.9	0.714
TOTPWR	18224	8902	11002	16124	23743	17498	12477	8919	13736	21744	17099	10273	9648	14338	24713	0.354
ULF	14919	8093	8797	12379	18709	14098	10139	7083	10675	18458	14311	8999	7867	11539	20217	0.368
VLF	1815	843	1184	1592	2384	1872	1417	961	1471	2416	1368	835	780	1254	1959	0.071
LF	861	522	481	711	1108	823	795	368	601	922	660	460	344	543	925	0.123
HF	629	502	293	471	743	705	715	205	496	815	760	602	251	527	1230	0.626
LF/HF	1.74	0.92	1.17	1.44	2.11	1.48	0.90	0.91	1.25	1.75	1.00	0.47	0.71	0.85	1.25	**<0.001**

St. Dev. standard deviation.

The p-values refer to Kruskal-Wallis test.

**Table 4:** Characteristics of the study sample of patients stratified according to IMT

	**Normal**	**Thickening**	**Plaque**	**P Values**			
				**ANOVA or χ**^**2**^	**Normal vs Thickening**	**Normal vs Plaque**	**Thickening vs Plaque**
Age (years)	47.3 ± 13.3	57.0 ± 9.6	65.9 ± 9.8	**<0.001**	**0.004**	**<0.001**	**0.001**
Sex (male/female)	16/10	10/12	101/51	0.157			
Family history of hypertension (yes/no)	16/10	18/4	79/73	**0.026**			
Family history of stroke (yes/no)	4/22	6/16	29/123	0.564			
Smokers (yes/ex/no)	4/3/19	6/2/14	25/37/90	0.242			
Diabetes (yes/no)	2/24	1/21	17/135	0.572			
Diastolic BP (mmHg)	76.5 ± 10.8	73.4 ± 18.6	75.8 ± 11.1	0.643	1	1	1
Systolic BP (mmHg)	131.5 ± 19.2	124.1 ± 33.2	134.8 ± 21.1	0.106	0.768	1	0.113
Pulse pressure (mmHg)	55.0 ± 14.9	50.6 ± 19.9	59.0 ± 17.8	0.086	1	0.856	0.117
Fasting blood glucose (mmHg)	98.0 ± 20.7	95.7 ± 22.0	104.5 ± 24.7	0.154	1	0.601	0.321
Total Cholesterol (mg/dl)	182.0 ± 38.0	192.0 ± 39.1	185.6 ± 41.4	0.687	1	1	1
BMI (kg/m^2^)	28.8 ± 5.4	27.3 ± 3.7	27.6 ± 4.6	0.395	0.747	0.596	1
Beta-blockers (yes/no)	15/11	6/16	46/106	**0.019**			
Alphabeta-blockers (yes/no)	1/25	3/19	17/135	0.465			
Alpha-blockers (yes/no)	1/25	2/20	13/139	0.702			
Diuretics (yes/no)	9/17	8/14	70/82	0.429			
ACE inhibitor (yes/no)	10/16	8/14	57/95	0.989			
Dihydropyridine (yes/no)	7/19	4/18	41/111	0.675			
eGFR (mL/min/1.73 m^2^)	84.1 ± 17.5	81.2 ± 17.8	75.2 ± 19.1	0.045	1	0.077	0.479
Renal involvement	9/15/2	6/13/3	33/91/28	0.502			
IMT (mm)	0.89 ± 0.11	1.19 ± 0.08	2.62 ± 1.61	**<0.001**	1	**<0.001**	**<0.001**
LVMi g/m^2^	109.6 ± 19.0	126.7 ± 33.2	134.6 ± 30.9	**<0.001**	0.149	**<0.001**	0.744
Left Ventricular hypertrophy (no/yes)	20/6	11/11	49/103	**<0.001**			

Data are expressed as mean and standard deviation for continuous variables (i.e. age) and as number of patients per each group for categorical variable (i.e. gender).

The p-value refers to ANOVA and post-hoc comparison with Bonferroni corrections for continuous variables and to χ^2^ for categorical variables.

**Table 5:** Comparisons of HRV measurement in the group of patients stratified by IMT

	**Normal IMT**					**Thickening**					**Plaque**					**P-value**
	**Mean**	**St. Dev.**	**Quartiles**			**Mean**	**St. Dev.**	**Quartiles**			**Mean**	**St. Dev.**	**Quartiles**			
			**1**^**st**^	**2**^**nd**^	**3**^**rd**^			**1**^**st**^	**2**^**nd**^	**3**^**rd**^			**1**^**st**^	**2**^**nd**^	**3**^**rd**^	
AVNN	830.0	123.5	749.2	821.6	916.2	842.3	141.8	746.7	820.5	957.3	879.0	124.7	797.1	864.8	952.0	0.116
SDNN	140.1	48.8	105.3	132.3	171.3	121.7	34.3	94.2	115.3	147.2	116.9	33.3	93.3	111.6	136.9	**0.049**
SDANN	128.4	49.7	88.2	124.8	157.9	110.8	34.7	81.0	102.2	138.3	104.1	33.5	80.3	98.5	124.1	**0.046**
SDNN IDX	58.9	19.0	44.4	55.2	73.1	50.7	13.8	43.0	48.9	56.5	49.0	14.2	38.8	47.4	59.5	**0.042**
RMSSD	36.9	13.5	26.8	32.2	42.6	30.7	11.7	22.1	29.7	34.0	35.2	17.9	22.7	30.5	41.7	0.216
pNN50	12.5	9.1	5.9	8.2	16.5	9.2	9.1	2.8	6.1	11.3	10.6	10.0	3.1	8.3	15.5	0.195
TOTPWR	24209	16727	12113	19664	31647	17583	10891	9798	14497	22534	16480	9863	9300	13860	21255	**0.049**
ULF	19550	13805	9436	16075	25947	14453	9137	7958	11822	18562	13420	8327	7216	10942	17851	0.092
VLF	2590	1944	1113	2117	3172	1746	1101	1091	1514	1862	1640	1027	960	1325	2251	0.052
LF	1255	1040	471	1006	1863	819	627	493	688	934	726	595	357	568	934	**0.009**
HF	813	685	305	542	1299	566	517	178	499	617	694	661	229	477	914	0.376
LF/HF	1.78	0.98	1.05	1.53	2.25	2.02	1.25	1.14	1.55	2.71	1.33	0.75	0.82	1.13	1.68	**0.002**

St. Dev. standard deviation.

The p-values refer to Kruskal-Wallis test.

**Table 6:** Characteristics of the study sample of patients with and without LVH

	**LVH**		**P values**
	**Patient Group with LVH**	**Patient group without LVH**	
Age (years)	56.8 ± 12.1	66.3 ± 10.6	**0.001**
Sex (male/female)	49/31	78/42	0.589
Family history of hypertension (yes/no)	48/32	65/55	0.415
Family history of stroke (yes/no)	15/65	24/96	0.827
Smokers (yes/ex/no)	17/18/45	18/24/78	0.404
Diabetes (yes/no)	2/78	18/102	**0.004**
Diastolic BP (mmHg)	73.8 ± 12.1	76.8 ± 11.8	0.094
Systolic BP (mmHg)	126.1 ± 25.3	137.9 ± 19.4	**0.001**
Pulse pressure (mmHg)	52.3 ± 18.0	61.1 ± 16.8	**0.001**
Fasting blood glucose (mmHg)	99.8 ± 23.6	104.7 ± 24.3	0.161
Total Cholesterol (mg/dl)	191.0 ± 38.6	182.3 ± 41.7	0.132
BMI (kg/m^2^)	27.2 ± 4.3	28.0 ± 4.8	0.235
Beta-blockers (yes/no)	31/49	36/84	0.199
Alphabeta-blockers (yes/no)	7/73	14/106	0.510
Alpha-blockers (yes/no)	5/75	11/109	0.456
Diuretics (yes/no)	25/55	62/58	**0.004**
ACE inhibitor (yes/no)	25/55	50/70	0.136
Dihydropyridine (yes/no)	17/63	35/85	0.211
eGFR (mL/min/1.73 m^2^)	79.9 ± 17.9	75.1 ± 19.5	0.071
Kidney Involvement (1/2 /3)	24/44/12	24/75/21	0.268
IMT (mm)	1.82 ± 0.93	2.52 ± 1.82	**<0.001**
Vascular abnormalities (no/ thickening/plaque)	20/11/49	6/11/103	**<0.001**
LVMi g/m^2^	105.0 ± 13.8	147.5 ± 27.3	**<0.001**

Data are expressed as mean and standard deviation for continuous variables (i.e. age) and as number of patients per each group for categorical variable (i.e. gender).

The p-values refer to t-test for continuous variables and χ^2^ for categorical variables.

**Table 7:** Comparisons of HRV measurement in the group of patients with and without LVH

	**Patients with LVH**					**Patients without LVH**					**P values**
	**Mean**	**St. Dev.**	**Quartiles**			**Mean**	**St. Dev.**	**Quartiles**			
			**1**^**st**^	**2**^**nd**^	**3**^**rd**^			**1**^**st**^	**2**^**nd**^	**3**^**rd**^	
AVNN	846.9	121.8	772.6	829.5	916.8	883.1	129.2	789.9	863.7	963.8	0.071
SDNN	123.5	37.0	101.5	118.3	142.3	118.4	36.0	91.8	112.7	142.4	0.247
SDANN	111.2	37.6	88.0	106.7	128.7	105.9	36.3	79.4	99.0	129.5	0.304
SDNN IDX	51.9	15.0	42.7	49.5	60.8	49.5	15.2	38.5	47.8	58.4	0.127
RMSSD	32.8	14.5	22.9	29.2	36.4	36.4	18.1	23.1	32.4	42.3	0.123
pNN50	9.9	10.3	3.2	7.1	11.9	11.2	9.4	3.4	9.2	17.0	0.163
TOTPWR	18472	12108	10873	15952	21928	17029	10776	8958	14057	22697	0.288
ULF	15018	10082	8177	12029	18491	13872	9043	7093	11412	18656	0.347
VLF	1892	1255	1074	1668	2393	1697	1206	923	1336	2086	0.105
LF	896	782	429	704	1092	744	619	369	556	912	0.072
HF	666	694	210	483	757	716	622	261	502	965	0.316
LF/HF	1.74	0.99	1.03	1.60	2.08	1.28	0.74	0.81	1.03	1.51	**<0.001**

St. Dev. standard deviation.

The p-values refer to Wilcoxon test.

**Table 8:** Multinomial or binary logistic regression models

**Compared groups**	**HRV measure, factor or covariate**	**β**	**p**	**OR**	**95% CI of OR**		
Normal eGFR versus Moderate decreased eGFR	Intercept	5.971	0.021				
	LF/HF	1.000	**0.038**	2.718	1.057	to	6.984
	Systolic BP	−0.003	0.779	0.997	0.974	to	1.020
	Age	−0.109	**<0.001**	0.897	0.847	to	0.948
	Absence of family history of hypertension	1.082	**0.047**	2.951	1.014	to	8.588
Mild decreased eGFR versus Moderate decreased eGFR	Intercept	0.527	0.819				
	LF/HF	0.969	**0.034**	2.637	1.075	to	6.465
	Systolic BP	0.024	**0.027**	1.024	1.003	to	1.046
	Age	−0.058	**0.020**	0.943	0.898	to	0.991
	Absence of family history of hypertension	0.710	0.122	2.034	0.827	to	5.001
Normal IMT group versus Plaque group^1^	Intercept	5.552	**0.002**				
	SDNN	0.018	**0.024**	1.018	1.002	to	1.033
	Age	−0.169	**<0.001**	0.844	0.796	to	0.896
	Absence of family history of hypertension	0.347	0.532	1.415	0.476	to	4.211
Thickening group versus Plaque group^1^	Intercept	2.688	0.101				
	SDNN	0.005	0.446	1.005	0.992	to	1.019
	Age	−0.080	**0.001**	0.923	0.879	to	0.970
	Absence of family history of hypertension	−1.072	0.071	0.342	0.107	to	1.096
Normal IMT group versus Plaque group^1^	Intercept	5.777	0.001				
	SDANN	0.017	**0.021**	1.017	1.003	to	1.032
	Age	−0.169	**<0.001**	0.845	0.797	to	0.696
	Absence of family history of hypertension	0.357	0.523	1.429	0.478	to	4.270
Thickening group versus Plaque group^1^	Intercept	2.626	0.101				
	SDANN	0.006	0.351	1.006	0.993	to	1.020
	Age	−0.080	**0.001**	0.923	0.879	to	0.970
	Absence of family history of hypertension	−1.059	0.075	0.347	0.108	to	1.112
group with Hypertrophy versus group without Hypertrophy^2^	Intercept	−5.240	**0.004**	0.005			
	LF/HF	−0.155	0.528	0.856	0.563	to	1.302
	Age	0.068	**<0.001**	1.071	1.036	to	1.107
	Cholesterol	−0.006	**0.141**	0.994	0.986	to	1.002
	Diuretics	−0.854	**0.011**	0.426	0.220	To	0.823

β: regression coefficient.

p: p-value referred to each variable in the regressions (multinomial or binary logistic).

OR: odds ratio, which is exp(β).

95% CI: 95% confidence interval.

^1^ multinomial logistic regression.

^2^ binary logistic regression.

## Discussion

To our knowledge, the current study is the first one to investigate standard linear HRV measures, both in time and in frequency domains, in hypertensive patients, categorised by severity of TOD at different levels (cardiac, vascular and renal) and considering adjusted models for age and other clinical parameters (such as gender, metabolic variables). Time and power spectral analysis of 24-hour electrocardiographic monitoring was performed in 200 hypertensive patients in basal conditions. At these same times, patients underwent echocardiographic and carotid ultrasonography study evaluations and routine laboratory evaluations in order to assess TOD at different level. The data of the current study showed that significantly lower values of some HRV measures were found in patients with CV involvement at different levels. Particularly, decreased LF/HF, which is a marker of sympatho-vagal balance, was found in the patients with moderate decreased eGFR, the highest grade of kidney involvement considered in this study. Similarly, depressed values of indexes of the overall ANS modulation on the heart, such SDNN and SDANN, were found in patients with plaque compared to patients with a normal IMT. These associations remained significant after adjustment for other factors known to contribute to the development of TOD, including diabetes, hypertension, and metabolic variables. Moreover, depressed LF/HF was found also in patients with LVH but this association was not significant after adjustment for other factors. As stated in a recent paper by Garcia-Garcia [23] the association between HRV and the development of TOD or vascular alterations has not been clearly established. Garcia-Garcia [23] investigated the association between 24-hours heart rate and IMT, LVMi, and eGFR, by performing a cross-sectional study including 360 hypertensive patients without heart rate lowering drugs, aged 30–80 years. We underline that they computed only two HR parameters: the mean HR and the standard deviation of HR (which are similar to AVNN and SDNN computed in the current study, respectively), while in this study almost all the linear HRV measures were computed as recommended and standardised in International Guidelines [8] and confirmed by recent literature [9]. Moreover, we proposed a larger number of covariates than Garcia-Garcia [23] in the adjusted model, for instance, the familiarity for hypertension or stroke. They found no association between AVNN and SDNN with LVH, consistently with our results. Moreover, they found associations between AVNN and SDNN with respect to IMT and eGFR, but these were lost after adjusting for age. Our results were coherent with these findings, even if in our studies the association of SDNN with IMT was significant also after adjustment. However, the patients in the study by Garcia-Garcia [23] were younger (56 ± 11 years versus 62 ± 12 years) and with a lower percentage of heart (18% vs 60%), and vascular (23% vs 75%) TOD. Moreover, the HRV analysis in this study was performed after a one-month antihypertensive therapy wash-out while many patients in the sample studied by Garcia-Garcia received drug therapy (not HR lowering drugs). We underline that the therapy before ECG holter measurement among selected groups was comparable as no significant differences occurred. The only exception was represented by diuretics, as a larger proportion of patients in the moderate eGFR group assumed diuretics compared with the other two groups (mild and normal eGFR) and a larger proportion of patients in the hypertrophy group assumed them compared to the group without hypertrophy. This difference was due to the fact that the antihypertensive treatment of patients with moderate decreased eGFR or with hypertrophy was based on the combination of diuretics with other drugs (i.e. Beta-blockers, ACE inhibitor, AT1 Antagonist and Dihydropyridine) which were usually prescribed also in the other groups of patients. We underline that the association of diuretics with a lower HRV has been already shown by the ARIC study [41] in 3577 hypertensive patients and by a recent study [42] in general male population, for that reason we performed the HRV analysis after a drug washout. The comparison of our results with those by Garcia-Garcia re-enforces the importance of computing several HRV parameters, especially the frequency-domain measures, such as LF/HF, which is considered as a non-invasive marker of the sympatho-vagal balance. However, the plausible mechanisms by which abnormal autonomic balance may lead to TOD and, particularly, renal organ damage one are not clearly known. Our results were consistent with two recent studies [20,21], investigating HRV and kidney disease, which concluded that lower HRV (particularly, frequency domain measures) was associated with higher risk of progression to end-stage renal disease and suggested that autonomic imbalance may lead to kidney damage. For that reason, prospective longitudinal studies are needed to evaluate a causal effect between HRV and TOD. In future studies, additional HRV measures, derived from nonlinear [43] and/or point process time-frequency [44] analysis, could be selected either in short-term recordings under standardised conditions [45] or in long-term continuous monitoring [46]. Moreover, other non-invasive parameters related to ANS activity, such as pupillometric features [47,48], could be adopted to provide additional information on autonomic cardiac control. As regards the other factors which entered the adjusted models, the age is the most significant variable, confirming that cardiac, vascular, and renal abnormalities increase progressively with age [49,50]. The absence of hypertensive familiarity seems to be associated with no renal involvement. Significantly higher systolic BP values were found in the mild decreased eGFR group, maybe because the blood pressure values are controlled in patients with moderate renal eGFR by the (significantly higher) use of diuretics. Higher systolic BP values were associated with LVH, coherently with the fact that it is an established cardiac manifestation of chronic hypertension [51]. Moreover, other factors associated with LVH are higher assumption rate of diuretics and higher values of cholesterol. Although previous studies have shown that gender influences HRV parameters both in healthy subjects [52] and hypertensive patients [53], this factor entered no final adjusted models. This means that within the selected sample of patients the gender-related differences in HRV were smaller than differences related to other factors and covariates, in particular age or progression of TOD. We consider in any case that the main limitations of this study were the inherent ones of observational and cross-sectional design, which precludes longitudinal analysis between HRV and TOD.

## Conclusions

In conclusion, depressed HRV appeared to be associated with vascular and renal TOD. In particular, depressed values of indexes of the overall ANS modulation on heart were found in patients with plaque compared to those with a normal IMT. These associations remained significant after adjustment for other factors known to contribute to the development of TOD. Moreover LF/HF, a marker of sympatho-vagal balance, was significantly decreased in the groups with mild and moderate decreased eGFR, confirming the involvement of autonomic imbalance in TOD. However, as the mechanisms by which abnormal autonomic balance may lead to TOD are not clearly known, we suggested that further prospective studies with longitudinal design would be performed to investigate HRV in the early stages of hypertension and of TOD development.

## Abbreviations

AVNN: Average of all NN intervals; BP: Blood Pressure; CI: Confidence Interval; CV: Cardiovascular; ECG: Electrocardiogram; eGFR: estimated Glomerular Filtration Rate; HF: Spectral power of all NN intervals between 0.15 and 0.4 Hz; HR: Heart Rate; HRV: Heart Rate Variability; IMT: Maximum arterial Intima Media Thickness; LF: Spectral power of all NN intervals between 0.04 and 0.15 Hz; LF/HF: Ratio of low to high frequency power; LV: Left Ventricle; LVH: Left Ventricular Hypertrophy; LVMi: LV Mass index; MDRD: Modification of Diet in Renal Disease; OR: Odds Ratio; pNN50: Percentage of differences between adjacent NN intervals that are longer than 50 ms; PSD: Power Spectral Density; RMSSD: Square root of the mean of the sum of the squares of differences between adjacent NN intervals; SDANN: Standard deviation of the averages of NN intervals in all 5-min segments of a 24-h recording; SDNN: Standard deviation of all NN intervals; SDNN IDX: Mean of the standard deviations of NN intervals in all 5-min segments of a 24-h recording; TOD: Target Organ Damage; TOTPWR: Total spectral power of all NN intervals up to 0.4 Hz; ULF: Spectral power of all NN intervals between 0 and 0.003 Hz; VLF: Spectral power of all NN intervals between 0.003 and 0.04 Hz.

## Competing interests

The authors declare that they have no competing interests.

## Authors’ contributions

PM participated in study conception and design, analysed data, contributed to interpretation of data and drafted the manuscript. RI collected the data, contributed to interpretation of data and revised critically the manuscript. NDL conceived the study, participated in its design and interpretation of data and helped to draft the manuscript. LP participated in study conception and design, data analysis and interpretation, helped to draft the manuscript. All authors read and approved the final manuscript.

## Authors’ information

PM and LP are biomedical engineers with research interest in biomedical signal processing for cardiologic risk assessment, particularly, by Heart Rate Variability analysis and using artificial intelligence methods. PM acquired ECTS-credits in the field of medicine, particularly cardiology. Moreover, PM and LP got their Ph.D. at the Faculty of Medicine of the University of Naples Federico II (in partnership with the Department of Biomedical, Electronic and Telecommunication Engineering of the same University). RI is a researcher in cardiologic science. NDL is an associate professor in cardiologic science.

## Pre-publication history

The pre-publication history for this paper can be accessed here:

http://www.biomedcentral.com/1471-2261/12/105/prepub
